# Beyond Kinase Activity: ERK5 Nucleo-Cytoplasmic Shuttling as a Novel Target for Anticancer Therapy

**DOI:** 10.3390/ijms21030938

**Published:** 2020-01-31

**Authors:** Alessandro Tubita, Zoe Lombardi, Ignazia Tusa, Persio Dello Sbarba, Elisabetta Rovida

**Affiliations:** Department of Experimental and Clinical Biomedical Sciences “Mario Serio”, University of Florence, 50134 Florence, Italy; alessandro.tubita@unifi.it (A.T.); zoe.lombardi@stud.unifi.it (Z.L.); ignazia.tusa@unifi.it (I.T.); persio@unifi.it (P.D.S.)

**Keywords:** nuclear localization, nuclear signaling, MAPK, BMK1, MAPK7, alternative kinase targeting, SUMOylation, protein phosphorylation, ubiquitination, chaperones

## Abstract

The importance of mitogen-activated protein kinases (MAPK) in human pathology is underlined by the relevance of abnormalities of MAPK-related signaling pathways to a number of different diseases, including inflammatory disorders and cancer. One of the key events in MAPK signaling, especially with respect to pro-proliferative effects that are crucial for the onset and progression of cancer, is MAPK nuclear translocation and its role in the regulation of gene expression. The extracellular signal-regulated kinase 5 (ERK5) is the most recently discovered classical MAPK and it is emerging as a possible target for cancer treatment. The bigger size of ERK5 when compared to other MAPK enables multiple levels of regulation of its expression and activity. In particular, the phosphorylation of kinase domain and C-terminus, as well as post-translational modifications and chaperone binding, are involved in ERK5 regulation. Likewise, different mechanisms control ERK5 nucleo-cytoplasmic shuttling, underscoring the key role of ERK5 in the nuclear compartment. In this review, we will focus on the mechanisms involved in ERK5 trafficking between cytoplasm and nucleus, and discuss how these processes might be exploited to design new strategies for cancer treatment.

## 1. Introduction

Protein phosphorylation is one of the key mechanisms used to transduce extracellular signals and transmit the information to the nucleus [1,2]. In particular, mitogen-activated protein kinases (MAPK) are a group of proteins able to translate environmental signals elicited by a plethora of stimuli, including growth factors and stresses, into different biological responses such as survival, apoptosis, proliferation and differentiation [3,4]. The importance of MAPK is underlined by the abnormal signaling conveyed by members of MAPK family in a number of human diseases, including Parkinson’s disease, inflammatory disorders and cancer [4,5]. There are several MAPK in mammals. The extracellular signal-regulated kinases 1 and 2 (ERK1/2), probably the best characterized among the classical MAPK, are activated mainly by growth factors, and are primarily involved in the transmission of proliferative signals. The c-Jun N-terminal kinase (JNK) 1/2/3 and p38MAPK α/β/γ/δ are activated mainly by inflammatory cytokines, and are primarily involved in adaptation to stress, apoptosis and differentiation. ERK5 (also known as Big MAPK 1, BMK1), the least characterized classical MAPK, is activated by both growth stimuli and stress, and plays critical roles in a number of cellular processes, including proliferation, differentiation and migration [6]. The MAPK family also includes the ERK3, ERK4, ERK7 atypical MAPK and the nemo-like kinase (NLK) [7].

MAPK pathways comprise a three-tier kinase cascade in which a MAPK is activated upon phosphorylation by a MAPK kinase (MAPKK), which in turn is activated when phosphorylated by a MAPKK kinase (MAPKKK) [8,9,10]. MAPK are evolutionarily well-conserved enzymes found in virtually all eukaryotes [11], and phosphorylate serine and threonine residues preceding a proline. Target specificity of each MAPK is determined by different docking domains [12]. These include the D-domain that consists of a conserved cluster of positively charged amino acids (a.a.) surrounded by hydrophobic ones, and is recognized by a short sequence of negatively charged a.a. of the C-terminus of MAPK, called the common docking (CD) domain. By tethering the MAPK to substrate, docking interactions contribute to the efficiency of kinase reaction [13,14]. This kind of regulation enhances the complexity of the MAPK signaling cascade and is responsible for the tuning of the wide variety of functional effects of MAPK family. Another important feature of MAPK signaling is the large number of cascade substrates, which include transcription factors, protein kinases and phosphatases, components of cytoskeleton, regulators of apoptosis, and a variety of other signaling-related molecules. Many of these substrates are localized in the nucleus, where they are involved in the regulation of transcription, while others are in the cytosol or cytoplasmic organelles and are responsible for processes such as translation, mitosis, apoptosis and migration [6].

## 2. Extracellular Signal-Regulated Kinase 5

ERK5 is expressed in many tissues, including heart, skeletal muscle, placenta, lung and kidney [15,16]. Discovered in 1995 independently by two research groups, ERK5 is encoded for by the *MAPK7* gene, which has a total length of 5824 bases and includes an open reading frame of 2451 bp. The gene product is a protein of 816 a.a. that has a two-fold molecular weight compared to the other classical MAPK family members, which explains why ERK5 was also given the name BMK1 [15].

Structurally, the ERK5 protein contains an N-terminal half (a.a. 1–406) endowed with kinase activity, and a C-terminal half of 410 a.a., important for the intracellular localization of ERK5 and the transcriptional regulation of target genes. The N-terminus includes a region required for cytoplasmic targeting (a.a. 1–77) and a kinase domain (a.a. 78–406) which shares 66% sequence identity to the kinase domain of ERK2. The kinase domain includes a region essential for the interaction of ERK5 with MEK5 (a.a. 78–139), an oligomerization domain (a.a. 140–406) and a CD domain (a.a. 350–358) important for the association with D-domain-containing substrates [17]. The C-terminal half includes a nuclear localization sequence (NLS) important for ERK5 nuclear targeting (a.a. 505–539), two proline-rich (PR) domains, namely PR1 (a.a. 434–465) and PR2 (a.a. 578–701), which are considered potential binding sites for Src-homology 3 (SH3)-domain-containing proteins, a nuclear export sequence (NES) and a myocyte enhancer factor 2 (MEF2)-interacting region (a.a. 440–501) [18]. The C-terminus of ERK5 also possesses a transcriptional transactivation domain (a.a. 664–789) [19] that undergoes autophosphorylation, thereby enabling ERK5 to directly regulate gene transcription, an ability unique to ERK5 amongst MAPK [20]. Furthermore, the C-terminus regulates ERK5 activation, autophosphorylation and nucleo-cytoplasmic shuttling (see below), and seems to have an auto-inhibitory function, as its truncation results in increased ERK5 kinase activity [21].

Initially identified as a stress MAPK, as it is activated by both oxidative and osmotic stresses [22], ERK5 was later shown to be also activated by a plethora of extracellular stimuli, including growth factors such as vascular endothelial growth factor (VEGF), epidermal growth factor (EGF), fibroblast growth factor-2 (FGF-2), platelet-derived growth factor (PDGF), colony-stimulating factor-1 (CSF-1), nerve growth factor (NGF), and interleukin 6 (IL-6) [23,24,25,26]. Furthermore, physiological and pathological conditions including laminar shear stress, ischemia and hypoxia are able to activate ERK5, although via mechanisms which still need to be fully elucidated [22,27,28,29]. Following activation by the above-listed stimuli, ERK5 controls cell survival and apoptosis, proliferation, differentiation, motility and angiogenesis [30,31,32,33]. Indeed, one of the first in vivo studies showed that ERK5 may support the viability of endothelial cells in adult animals [34], and is critical for embryogenesis, probably due to its role in the control of proliferation of endothelial cells and vasculogenesis [27,35,36].

How activated membrane receptors couple to ERK5 is still partially unclear. Growth factor receptors may cause ERK5 activation through Ras in certain cell types [37,38], but not in others [39]. In addition, other intracellular kinases, such as MEKK2/3 [40], c-Cot [41] and c-Src [42] may activate ERK5. Of note, the adaptor protein Lad1/RIBP by regulating MEKK2, but not MEKK3, is involved in ERK5 pathway activation [43]. Direct ERK5 activation is classically operated by the upstream MAPKK, MEK5, that has ERK5 as its only known substrate [15] and phosphorylates ERK5 at T218/Y220 in the conserved threonine-glutamic acid-tyrosine (TEY) motif of the catalytic domain [39]. MEK5-dependent phosphorylation contributes to ERK5 stabilization in an active conformation, the latter event being further promoted by ERK5 auto-phosphorylation at the C-terminus. Importantly, phosphorylation of the C-terminus is required for maximal ERK5 transactivator activity, which is exerted following its nuclear localization and the consequent phosphorylation of nuclear targets [21]. On the other hand, it has been shown that the nuclear localization of a mutant ERK5 form devoid of kinase activity results in the activation of transcription through the transcriptional activation domain (TAD) located at the C-terminus [44].

Like other MAPK, ERK5 phosphorylates its substrates at S/T-residues immediately preceding a proline. Intriguingly, residue T28 in the ERK5 N-terminal half and residues S421, S433, S496, S731 and T733 in the C-terminal half are not followed by proline, but undergo autophosphorylation. Furthermore, ERK5 is capable of phosphorylating MEK5 at specific proline-unrelated sites, the residues S129, S137, S142 and S149 [45]. Taken together, these findings suggest that the substrate specificity of ERK5 may differ from that of other MAPK family members. The best characterized ERK5 substrates are nuclear transcription factors, while the known ERK5 cytosolic substrates are very few and include p90RSK kinases [46], the pro-apoptotic protein BAD and the GAP junctional protein CX43 [47]. Although the direct phosphorylation of these substrates by ERK5 has not been demonstrated (except for CX43), it is a fact that ERK5 silencing reduces the phosphorylation of these proteins.

Once ERK5 has been activated, it translocates into the nucleus where it phosphorylates and activates a number of transcription factors, of which the MEF-2 family members MEF2A, C and D are the best characterized [39,48,49]. In particular, ERK5 phosphorylates MEF2C in S387, thus increasing its transcriptional activity that in turn enhances c-Jun expression [50]. MEF2D has been shown to be an ERK5-specific substrate [48,49], whereas the activities of MEF2A and C are controlled by both ERK5 and p38 MAPK [51,52]. As mentioned above, ERK5 contains a MEF2-interacting region and a transcriptional transactivation domain in its C-terminus, both being critical to regulate MEF2 activity [19], as demonstrated by the fact that an ERK5 mutant lacking the C-terminus fails to stimulate MEF2 activity [18]. Besides regulating MEF2, ERK5 controls the transcription of c-MYC, CREB and Sap1a [38,53]. Furthermore, it has been shown that, while both ERK5 and ERK1/2 are capable to phosphorylate c-Fos at S387, ERK5 activation determines c-Fos phosphorylation at additional sites, leading to maximal c-Fos transactivation activity; phosphorylation of these c-Fos sites requires the C-terminal tail of ERK5 [54]. Finally, ERK5 activates other transcription factors, such as peroxisome proliferator-activated receptor delta (PPARδ) [55] and probably PPARγ [56] and nuclear factor κB (NFκB) [57]. Importantly, ERK5 possesses an intrinsic transcriptional transactivation activity, which was demonstrated to induce the transcription of *Nur77* gene upon calcium signals in T cells [19].

With respect to other downstream pathways of ERK5, it has been reported that it plays a relevant role in regulating cell cycle progression [58], and that there is a link between ERK5 and NFκB in the regulation of cell cycle through the control of G2-M transition and timely entry into mitosis [59]. This function requires ERK5-dependent activation of NFκB via ribosomal S6 kinase 2. Moreover, during mitosis, ERK5 is constitutively phosphorylated and binds and inactivates BIM, a BCL2 family mediator of cell death, suggesting that ERK5 plays a role in the survival of cells in mitosis [60].

## 3. Mechanisms of Regulation of ERK5 Nuclear Translocation

**Figure 1:**
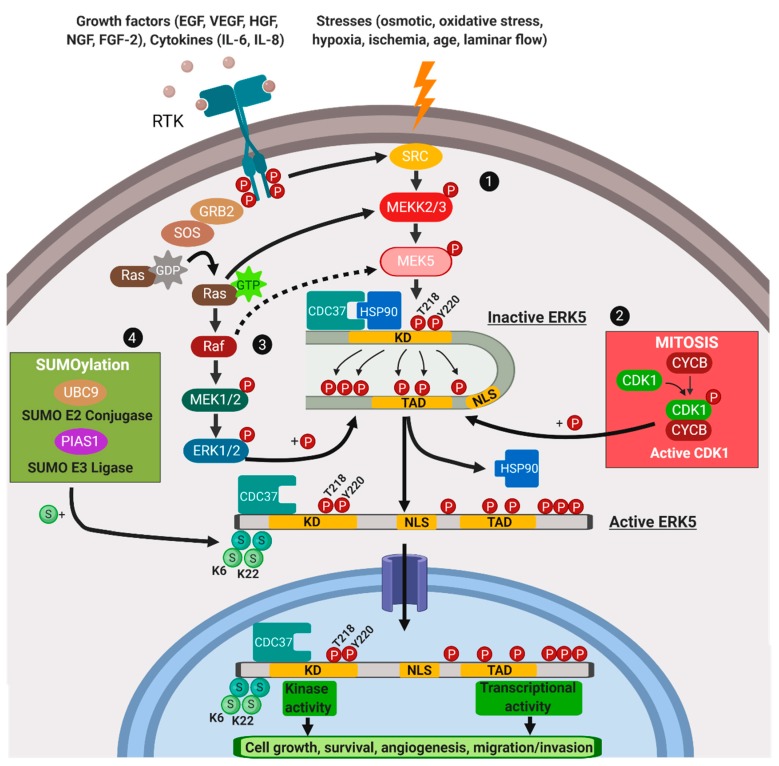
ERK5 nuclear translocation. In the inactive state, the N-terminal half of ERK5 interacts intra-molecularly with the C-terminal half, and the chaperones HSP90 and CDC37 are bound to ERK5. In this conformation ERK5 resides in the cytosol. (1) Upon MEK5-mediated phosphorylation of the TEY motif and subsequent activation, ERK5 auto-phosphorylates its C-terminal half determining the intramolecular interaction, and inducing a conformational change that results in the dissociation from HSP90, exposition of the NLS, and nuclear translocation. Alternatively, MEK5-independent mechanisms resulting in ERK5 phosphorylation and nuclear translocation include: (2) phosphorylation by CDK1 at S753 and/or T732 in mitosis; (3) MEK1/2-ERK1/2-dependent mechanism, involving ERK5 phosphorylation at T732, under growth factors stimulation and/or oncogene activation. (4) Regulation of ERK5 nuclear shuttling by SUMOylation at the N-terminus. Solid lines indicate direct established regulatory interactions, whereas broken lines illustrate putative interactions (created with Biorender.com).

As reported above, many MAPK exert their ultimate activities in the nucleus. Translocation of MAPK from the cytosol to the nucleus is indeed essential for the regulation of gene transcription and cellular processes such as cell cycle progression, differentiation and circadian clocks [61,62]. Small molecules including proteins can enter the nucleus by simple diffusion through the nuclear pore, while proteins with a molecular mass larger than around 60 kDa, including many MAPK, are actively transported from one side to the other of the nuclear envelope by nuclear transporters. Regarding the latter, for example, ERK1/2 are shuttled by Importin-7 (Imp7) [63,64], while JNK and p38 are shuttled by a dimer of Imp3 with either Imp9 or Imp7 [65]. With respect to ERK5 nuclear translocation, several mechanisms of regulation have been described (Figure 1).

ERK5 is expressed in a number of cell types, and its intracellular localization under routine culture conditions varies largely [21], ranging from a predominantly nuclear pattern, such as that in COS-7, HeLa, BT474 and SKBR3 cells [50,66,67], to an overall diffuse pattern, like in MCF7 cells [66]. In the murine myoblast cell line C2C12 and the breast cancer cell line MCF7, ERK5 localizes in the cytoplasm as a result of serum deprivation and translocates into the nucleus in response to FGF or neuregulin [50,66]. In HeLa cells, ERK5 is localized in the nucleus even in the absence of stimulation, but treatment with EGF causes a further increase of nuclear ERK5 [50,67]. Furthermore, following EGF-induced nuclear translocation, ERK5 is activated by nuclear MEK5, after this kinase is activated, in turn, by nuclear MEKK2 [67]. More recently, it was reported that EGF-induced MEKK2 nuclear translocation is affected by calcium levels, so that low as well as high calcium levels reduce ERK5 activity in the nucleus [68]. Other growth factors reported to induce ERK5 nuclear translocation are CSF-1 in murine macrophages [69] and PDGF in human hepatic stellate cells [70]. Finally, activated Src as well as mutated BRAF (BRAFV600E) cause ERK5 nuclear translocation [37,71].

The presence within ERK5 of a large C-terminal tail raises questions about its potential role in affecting ERK5 signaling. Buschbeck and colleagues showed that C-terminal half influences not only ERK5 activation but also its nuclear shuttling [21]. Indeed, deletion of the last one hundred C-terminal residues (ERK5∆713) not only leads to a marked increase of ERK5 kinase activity, probably due to the fact that the C-terminal tail possesses an autoinhibitory function of kinase activity [53], but also results in ERK5 nuclear accumulation. Further deletion of most or all of the ERK5 C-terminal half (ERK5∆575, ERK5∆464 and ERK5∆409) in COS-7 cells results in the loss of its predominant nuclear localization and in an equal distribution between the cytosol and the nucleus. Such an altered distribution of differently truncated ERK5 forms can be explained by the presence of a functional NLS (a.a. 505–539) and a NES (a.a. 440–501) in the C-terminal half of ERK5 [18]. This is in agreement with the cytoplasmic accumulation of a murine ERK5 variant truncated in the C-terminus (mERK5-t) after residue 492 [72]. Additional lines of evidence linked nuclear ERK5 to a pro-tumoral effect. Indeed, in HeLa cells, the expression of another truncated ERK5 form (ERK5∆570), which resides in the nucleus constitutively, reduces apoptotic cell death in response to TRAIL receptor activation [44]. Interestingly, ERK5 nuclear localization has been proposed to be an early event in the onset of hepatocellular carcinoma [70], and a strong nuclear ERK5 expression is associated with a relatively poor prognosis of prostate cancer [73]. Later work showed that Mir143 has a tumor suppressor role in prostate cancer by controlling cell proliferation and survival through modulation of ERK5 [74], and that Mir143 expression inversely correlates with nuclear ERK5 immunoreactivity in clinical prostate cancer [75].

### 3.1. MEK5-Dependent Nuclear Translocation of ERK5

Under basal conditions, i.e., in unstimulated cells and/or in the absence of oncogenic stimuli, cytosolic ERK5 is in an unphosphorylated inactive folded form, where the N- and the C-terminal halves are bound to each other, so that the NLS is hidden and nuclear translocation is prevented [18]. This conformation is stabilized by the interaction of ERK5 with the co-chaperone CDC37 and the chaperone HSP90, the latter ensuring cytosolic anchorage of ERK5 protein. Besides stabilizing ERK5 in an inactive conformation, the trimeric complex ERK5-CDC37-HSP90 facilitates ERK5 recognition and activation by MEK5 [76]. In the folded structure, the C-terminus masks the CD domain of the N-terminus, preventing ERK5 interaction with its substrates. MEK5-dependent phosphorylation at the TEY region initiates the kinase activity of ERK5, which can phosphorylate itself in the C-terminus, thereby promoting HSP90 release from the complex. Furthermore, following phosphorylation of the C-terminus, ERK5 may assume an open conformation, exposing the NLS sequence that allows ERK5 nuclear translocation [76,77]. The latter event likely involves Impα/β [78], that transports NLS-containing proteins across the nuclear envelope [79,80]. Finally, a mutated form of ERK5 that cannot be phosphorylated by MEK5 (ERK5-AEF, where TEY has been mutated to AEF) as well as ERK5∆713-AEF are unable to translocate into the nucleus upon stimulation [21,66]. Thus, phosphorylation of ERK5 at the MEK5 consensus site seems necessary for nuclear translocation, at least under certain conditions (see below). Once in the nucleus, ERK5 enhances gene transcription either by phosphorylating transcription factors or, in a kinase-independent manner, by interacting with transcription factors through the TAD domain.

### 3.2. MEK5-Independent Nuclear Translocation of ERK5

Besides MEK5-dependent activation, other mechanisms driving ERK5 nuclear translocation have been described. Among these, the overexpression of CDC37 in cancer cell lines induces HSP90 dissociation from ERK5, and nuclear translocation of wild type ERK5 as well as of a kinase-inactive form (D200A) which retains transactivation activity [76]. As stated above, ERK5 nuclear shuttling requires phosphorylation at the C-terminal half. This event may be promoted by ERK5 itself or by other kinases. Referring to the latter occurrence, it has been reported that four residues (S753, T732, S773, S706) of ERK5 C-terminal half may be phosphorylated during mitosis in a cyclin-dependent kinase (CDK)1-dependent manner, and that this phosphorylation is important for ERK5 nuclear localization [81]. Whether this phosphorylation determines NLS exposure, like in the case of MEK5-dependent phosphorylation, has not been clarified. On the other hand, this study could not conclude that CDK1 is the unique kinase phosphorylating ERK5 during mitosis, as only two out of the four identified phosphorylated ERK5 residues, S706 and T732, are followed by a proline and are CDK1 consensus target sites.

CDK5 is an unusual member of the CDK family, endowed with functions not related to cell cycle control, and, unlike other mitotic CDK, is activated by binding to p35 or p39 [82]. Moreover, CDK5 plays a relevant role in tumorigenesis in a number of cancers, such as breast, pancreas and neuroendocrine thyroid carcinomas [83,84,85], and has been recently demonstrated to directly phosphorylate ERK5 in T732, enhancing ERK5 nuclear accumulation and modulating the oncogenic ERK5-AP1 axis in colorectal cancer [86].

ERK1/2 may phosphorylate ERK5, resulting in an additional MEK5-independent activation of ERK5. A recent study showed that a constitutively active RAS mutant (RASV12) resulted in ERK5 phosphorylation at T732. The involvement of ERK1/2 in this process was suggested by the fact that treatment with a MEK1/2 inhibitor (U0126) reduces ERK5 phosphorylation at T732. This event induces ERK5 nuclear localization and promotes ERK5-dependent transcription, without affecting the phosphorylation status at TEY or at other (S769/S773/S775) C-terminal residues [87]. Along this line, we recently reported that the overexpression of BRAFV600E in melanoma cells increases the nuclear amount of total and phosphorylated ERK5 at S753 and T732, indicating that oncogenic BRAF, likely via ERK1/2 and CDK1, enhances ERK5 functions as well as nuclear localization. More importantly, oncogenic BRAF increased chromatin-bound ERK5, and enhanced the ability of ERK5 to induce transcription activity of MEF2, demonstrating that BRAF can also influence the latter ERK5 function [88]. All the above-described MEK5-independent mechanisms result in the nuclear translocation of ERK5 which exerts a transcriptional transactivation activity independently of its kinase activity.

Beyond kinases and chaperones, ERK5 activity and nuclear translocation are regulated by other mechanisms. The protein Ser/Thr phosphatases PP1/PP2A [89] and protein tyrosine phosphatases (PTP) not only block ERK5 activation but prevent ERK5 translocation to the nucleus [90]. Finally, the dual-specificity protein phosphatases DUSP5 and DUSP6 regulate ERK5 dephosphorylation at TEY motif [91]. If these events are linked to ERK5 nuclear trafficking has not been addressed.

### 3.3. ERK5 Ubiquitination and Chaperone-Dependent Transport to the Nucleus 

Ubiquitination is a post-translational modification consisting of the attachment of ubiquitin peptides to a substrate through lysine-linked isopeptide bonds, and involves three steps: activation, conjugation and ligation, which are catalyzed by specific enzymes. The ubiquitin-activating enzyme (E1) binds ubiquitin in an ATP-dependent manner and transfers it to a ubiquitin-conjugating enzyme (E2) that, with the help of ubiquitin-protein ligases (E3), attaches ubiquitin to target proteins [92]. Ubiquitin consists of 76 a.a., including seven lysine residues (K6, K11, K27, K29, K33, K48, and K63), which can be conjugated with lysine residues of other ubiquitin molecules, forming poly-ubiquitin chains. Ubiquitination targets substrates to the proteasome [93], thereby regulating the degradation of non-functional (i.e., unfolded) or fast-turnover (such as cyclins) proteins. Ubiquitination can also be related to non-proteolytic effects. Indeed, while poly-ubiquitin chains linked through K48 of ubiquitin usually target a protein for degradation by the proteasome, those linked through other lysine residues may lead to proteolytic as well as non-proteolytic events [94]. With respect to the latter effect, it has been demonstrated that K63-linked poly-ubiquitination of the yes-associated protein (YAP), a transcriptional regulator involved in cell proliferation, promotes YAP cyto-nuclear translocation, transcriptional activity and growth-promoting functions. This non-proteolytic ubiquitination is mediated by the S-phase kinase-associated protein 2 (SKP2), that acts as an oncogenic E3 ligase [95].

ERK5 interacts with the HSP90-CDC37 chaperones in resting cells, and inhibition of HSP90 or CDC37, by geldanamycin or celastrol, respectively, results in ERK5 ubiquitination and degradation [76]. MEK5-dependent phosphorylation/activation of cytoplasmic ERK5 drives HSP90 dissociation from ERK5-CDC37-HSP90 trimeric complex and determines ERK5 nuclear translocation and transcription activation by a mechanism which requires ERK5 autophosphorylation at the C-terminus. Consistently, active ERK5 is no longer sensitive to HSP90 inhibitors [76].

### 3.4. Impact of SUMOylation on ERK5 Nuclear Translocation

Besides phosphorylation and ubiquitination, protein modification by SUMOylation has come into focus as an important regulator of intracellular signaling due to the transient nature of these modifications [96]. Like ubiquitination, SUMOylation consists in the covalent attachment of small proteins, the small ubiquitin-like modifier (SUMO) proteins (SUMO1-4), to the lateral chain of lysine residues in the substrate, and occurs in three steps (activation, conjugation and ligation) led by enzymes different from those involved in ubiquitination. The SUMOylation cascade contains a SUMO-activating enzyme (E1), which is necessary for the activation of SUMO precursors, a SUMO-conjugating enzyme (E2) and a number of SUMO ligases (E3). Cell proteins constantly undergo SUMOylation and de-SUMOylation: the enzymes primarily responsible for deconjugating SUMOylated proteins are the sentrin-specific proteases (SENP), of which SENP2 has been identified as the protease that removes SUMO from ERK5 [96].

SUMOylation regulates biological processes involved in survival, apoptosis, proliferation, differentiation and senescence, and is critically involved in cancer onset and progression. SUMOylation impacts on the function of a number of proteins by modifying their subcellular localization, protein partnering, DNA binding and transactivating functions [97,98]. This mechanism of regulation is different from that operated by ubiquitination, which mostly results in substrate degradation. It is well known that SUMOylated proteins and proteins expressing SUMO-binding motifs co-aggregate in sub-nuclear compartments called promyelocytic leukemia factor (PML) nuclear bodies (NB). PML-NB are among the regulators of transcription, genome integrity, apoptosis, reaction to viral infection and tumor suppression [99]. For example, SUMOylation of the liver receptor homologue-1 (LHR-1) induces the localization of LRH-1 in PML-NB, resulting in the inhibition of its transcriptional activity. On the contrary, when LRH-1 is de-SUMOylated, its interaction with other PML-NB components fails and LRH-1 can bind active chromatin domains [100]. Another example concerns the transcription factor Sp3, that is typically SUMOylated and localized at nuclear periphery and at nuclear dots in a repressed state. Upon de-SUMOylation, Sp3 is converted into a transcriptional activator with a diffuse nuclear localization [101].

SUMOylation may induce protein redistribution from the cytoplasm to the nucleus. For example, it has been demonstrated that SUMOylation of K195 in Flot-1 is important for its mitogen-stimulated translocation into the nucleus in PC3 cells [102]. Another example of SUMO-dependent cytosol to nucleus redistribution concerns the transcriptional corepressor C-terminal-binding protein 1 (CtBP1). It has been demonstrated that SUMOylation of CtBP1 at K48 results in its nuclear localization and the triggering of its corepressor function in the regulation of E-cadherin expression [103].

The first evidence that ERK5 may be SUMOylated has been reported by Woo and colleagues, by showing that ROS induce SUMOylation of endogenous ERK5 at K6 and K22, resulting in the inhibition of ERK5 transcriptional activity without affecting ERK5 phosphorylation and kinase activity in endothelial cells. Based on that, the authors hypothesized that the reduction of ERK5 transcriptional activity upon SUMOylation could be attributable to the increase of ERK5 interaction with repressors or the decrease of ERK5 interaction with co-activators [104]. This study did not directly address the question whether SUMOylation affects the amount of nuclear ERK5. More recently, Erazo and colleagues found that ERK5 SUMOylation supports ERK5 nuclear trafficking, and stimulates, rather than inhibiting, ERK5-mediated transcriptional activation and cancer cell proliferation (Tatiana Erazo, Sergio Espinosa-Gil, Nora Diéguez-Martinez, Nestor Gomez and Jose M Lizcano; submitted for publication in IJMS, Special Issue “Targeting MAPK in Cancer”).

### 3.5. Possible Role of ERK5 Dimerization/Oligomerization on its Nuclear Translocation

Protein dimerization is a well-established mechanism driving the transduction of extracellular signals. As far as the MAPK system is concerned, it has been shown in vitro that ERK2 phosphorylation facilitates the formation of ERK2 dimers and that dimerization is necessary for ERK2 localization to the nucleus [105]. ERK1 is also capable of dimerization when phosphorylated, both in vivo and in vitro [106]. It has been also reported that the N-terminal (a.a. 140–406), but not the C-terminal, half of ERK5 is involved in oligomerization. Moreover, while ERK1/2 oligomerizes upon phosphorylation, oligomerization of ERK5 is observed in both activated and control cells (with or without the expression of MEK5DD, a constitutively active form of MEK5), suggesting that ERK5 oligomerization does not depend on its phosphorylation status [18]. To the best of our knowledge, no data are available in literature about a possible impact of ERK5 dimerization/oligomerization on its nuclear translocation, so that further studies are needed to address this point.

### 3.6. Possible Impact of ERK5 Mutations on its Nuclear Translocation

*MAPK7* is mutated in a large variety of human cancers, although activating mutations of ERK5 have not been reported. We recently showed that five out of 479 melanoma patients harbor *MAPK7* missense mutations, including P789S and A424S, two potential phosphorylation targets of C-terminus worth being characterized for their possible impact on ERK5 nuclear shuttling [88]. In silico data analysis of 32 types of cancers in 10,953 patients (data from TCGA PanCancer Atlas Studies available from the cBioPortal) allowed us to establish that none of the reported MAPK7 missense mutations is involved in ERK5 SUMOylation or is located in the TEY motif. Interestingly, five out of six mutations found in the NLS sequence (R505W, R513C, R515L, R521W, R524W) determine the replacement of polar arginine residues, a stretch of which constitutes the NLS, with non-polar a.a.. This fact is unlikely pro-tumorigenic because it might result in the reduction, rather than the increase, of ERK5 nuclear translocation. With respect to the C-terminus, six mutations (P605S, P607S, P609S, A653T, G744S, P789S) result in the replacement of non-phosphorylatable residues with phosphorylatable ones. We may speculate that phosphorylation at these sites may favor ERK5 nuclear shuttling. On the other hand, none of the known phosphorylatable residues of the C-terminal tail which are primarily involved in ERK5 nuclear translocation, including S753 and T732, are mutated in cancer patients.

## 4. Concluding Remarks: Targeting ERK5 Cytoplasm-to-Nucleus Shuttling

The involvement of ERK5 in the pathogenesis and progression of several types of cancer is well established [107]. Accordingly, targeting the MEK5-ERK5 pathway is among the emerging strategies for cancer treatment [107,108,109]. Several small molecule inhibitors of ERK5 or MEK5 kinase activity have been developed (Table 1) and are very effective in reducing tumor growth in vitro and in vivo in a number of cancers. However, it is also emerging that the oncogenic role of ERK5 may be kinase inhibitor-insensitive [110]. A possible reason for this insensitivity is that ERK5 nuclear accumulation, a crucial event in sustaining cancer cell proliferation [77], is indeed often independent of the kinase activity of MEK5 or ERK5 itself as indicated by the ineffectiveness of ERK5 or MEK5 inhibitors when used as single agents. Indeed, ERK5 localizes in the nucleus of CLB-BAR and CLB-GE human neuroblastoma cell lines even in the presence of the ERK5 inhibitor XMD8-92 [111], and neither BIX02189 nor XMD8-92 are able to suppress ERK5 nuclear accumulation when used alone, but only in combination with vemurafenib, in melanoma cells expressing BRAFV600E [88]. Along this line, ERK5 and MEK5 inhibitors are expected to be effective in preventing ERK5 nuclear translocation when used in combination with ERK1/2- or CDK-targeting drugs. In this respect, the dual ERK5/CDK inhibitor TG02, tested in clinical trials for hematological malignancies following the promising results obtained in preclinical studies [112,113], may provide a desirable effect in this direction.

Based on all above, targeting ERK5 nuclear translocation provides new opportunities to inhibit ERK5 biological functions. For example, targeting SUMOylation as well as HSP90 may prevent ERK5 nuclear translocation [131,132], whereas the combination of ERK5 and HSP90 inhibitors was effective in vitro and in vivo against TNBC, leading to the upregulation of pro-apoptotic effectors [133]. However, whether this is linked to the reduction of ERK5 nuclear accumulation was not addressed. Another intriguing point to be further investigated is the possible involvement of phosphatases in ERK5 phosphorylation at the C-terminus, possibly providing additional opportunities for targeting ERK5 function. Finally, in case ERK5 di-/oligo-merization will be found necessary for its nuclear redistribution, inhibitors of this phenomenon should be developed.

Besides showing that ERK5 translocation into the nucleus is regulated by a bipartite NLS-dependent nuclear import mechanism, Kondoh and Colleagues provided evidence that ERK5 nuclear export is CRM1-dependent and is therefore blocked by leptomycin B [49]. On one hand, this suggests to further investigate whether nuclear export of ERK5 may be impaired in cancer cells, thus contributing to the increase of nuclear ERK5. On the other hand, restoration of nucleus-to-cytosol efflux should be sought after as an additional strategy to reduce the effects of ERK5 which depend on its nuclear localization.

Finally, another possible approach to prevent ERK5 nuclear translocation is the suppression of ERK5 protein via PROteolysisTArgetingChimeras (PROTACs), which are heterobifunctional small molecules that modulate protein target levels by hijacking the ubiquitin-proteasome system to induce degradation of the target. This approach would overcome the insensitivity to kinase inhibitors as well other inhibitor classes, resulting therefore particularly powerful in the suppression of the activity of non-druggable targets [134].

## Figures and Tables

**Table 1:** MEK5/ERK5 small molecule inhibitors. ^1^ Cell free in vitro assay; ^2^ *Kd* value determined using the BROMOscan assay (DiscoveRx); ^3^ IC50 value determined by AlphaScreen binding assay; ^4^ ClinicalTrials.gov Identifier: NCT01699152 (I), NCT01204164 (I), NCT03904628 (I), NCT03224104 (I), NCT03738111 (I), NCT02942264 (II); ^5^ *Kd* using an affinity steady-state 1:1 binding model; ^6^ ClinicalTrials.gov Identifier: NCT00033384; NCT00034827.

Inhibitor	Main Target	Enzymatic IC_50_^1^ (nM)	Other Targets (IC_50_ nM)	Mechanism of Action	Phase of Development	Ref.
XMD8-92	ERK5	364	LRRK2 (59), BRD4 (170 ^2^)	ATP-competitive	preclinical	[110,114,115]
XMD17-109 (ERK5-IN-1)	ERK5	162	LRRK2 (339), BRD4 (217 ^3^)	ATP-competitive	preclinical	[114,116]
JWG-045 (XMD10-78)	ERK5	98	BRD4, (11,000 ^3^) LRRK2 (289 ^3^)	ATP-competitive	preclinical	[116,117]
JWG-071	ERK5	88	BRD4 (5420 ^3^), LRRK2 (109 ^3^), DCAMKL2 (223 ^3^), PLK4 (328 ^3^),	ATP-competitive	preclinical	[116]
AX15836	ERK5	8	BRD4 (3600 ^2^)	ATP-competitive	preclinical	[110]
BAY-885	ERK5	35		ATP-competitive	preclinical	[118]
TG02 (SB1317, Zotiraciclib)	CDKs ERK5	3–37 43	JAK1 (59), JAK2 (19), FLT3 (19)		I/II ^4^	[112,119,120]
Compound 46	ERK5	820	BRD4 (no binding ^5^)		preclinical	[121,122,123]
SC-1-181	MEK5			ATP-competitive	preclinical	[122,123]
BIX02188	MEK5	4.3	ERK5 (810), CSF1R (280), LCK (390)	ATP-competitive	preclinical	[124]
BIX02189	MEK5	1.5	ERK5 (59), CSF1R (46), LCK (250)	ATP-competitive	preclinical	[124]
GW284543 (UNC10225170)	MEK5			ATP-competitive	preclinical	[125]
CI-1040 (PD184352)	MEK1/2	17	MEK5	ATP non-competitive	II ^6^	[126,127]
UO126	MEK1 MEK2	72 58	MEK5	ATP non-competitive	preclinical	[38,127,128]
PD98059	MEK1 MEK2	2–7 (μM) 50 (μM)	MEK5	ATP non-competitive	preclinical	[38,129,130]

## Abbreviations

BMK1	Big MAP Kinase 1
CD	Common Docking
CDK1	Cyclin-Dependent Kinase 1
CtBP1	C-terminal-Binding Protein 1
CSF-1	Colony-Stimulating Factor-1
DUSP	Dual-Specificity Protein Phosphatases
EGF	Epidermal Growth Factor
ERK1/2	Extracellular Signal-Regulated Kinase 1/2
ERK5	Extracellular Signal-Regulated Kinase 5
FGF-2	Fibroblast Growth Factor-2
IL-6	Interleukin 6
Imp	Importin
JNK	c-Jun N-terminal Kinase
LHR-1	Liver Receptor Homologue-1
MAPK	Mitogen-Activated Protein Kinase
MAPKK	MAPK Kinase
MAPKKK	MAPKK Kinase
MEF2	Myocyte Enhancer Factor 2
NB	Nuclear Bodies
NGF	Nerve Growth Factor
NES	Nuclear Export Sequence
NLK	Nemo-Like Kinase
NLS	Nuclear Localization Sequence
PDGF	Platelet-Derived Growth Factor
PML	Promyelocytic Leukemia Factor
PP1/PP2A	Protein Ser/Thr Phosphatases
PPARδ	Peroxisome Proliferator-Activated Receptor Delta
PR	Proline-Rich
PROTACs	PROteolysisTArgetingChimeras
PTPs	Tyrosine-Specific Phosphatases
SENP	Sentrin-Specific Protease
SH3	Src-Homology 3
SKP2	s-Phase Kinase-associated Protein 2
SUMO	Small Ubiquitin-like Modifier Proteins
TAD	Transcriptional Activation Domain
TEY	Threonine-Glutamic Acid-Tyrosine
VEGF	Vascular Endothelial Growth Factor
YAP	Yes-Associated Protein
